# An untargeted comparative metabolomics analysis of infants with and without late-onset breast milk jaundice

**DOI:** 10.1371/journal.pone.0308710

**Published:** 2024-08-12

**Authors:** Mingxuan Cui, Qianying Guo, Shilong Zhao, Xinran Liu, Chen Yang, Peng Liu, Linlin Wang

**Affiliations:** 1 Department of Clinical Nutrition, Peking University People’s Hospital, Beijing, China; 2 Institute of Reproductive and Child Health/National Health Commission Key Laboratory of Reproductive Health, School of Public Health, Peking University, Beijing, China; Fisheries and Oceans Canada, CANADA

## Abstract

Background: Late-onset breast milk jaundice (LBMJ) is a common form of hyperbilirubinemia, which can result in serious complications for newborns with persistently high bilirubin levels. The aim of this study was to investigate the differences in fecal metabolites between breastfed infants with and without LBMJ in order to elucidate potential biological mechanisms. Methods: Biological samples were collected from 12 infants with LBMJ and 12 healthy individuals. Ultra-high performance liquid chromatography quadrupole time-of-flight tandem mass spectrometry (UHPLC-Q-TOF/MS) was utilized for non-targeted determination of fecal metabolites. Principal components analysis (PCA), cluster analysis, and differential metabolite analysis were performed in both positive ion mode and negative ion mode for the two groups. Additionally, the KEGG database was employed to comprehensively analyze the pathways of differential metabolites. Results: There were no significant differences in maternal and neonatal demographic characteristics between the two groups (p > 0.05). The results of PCA and cluster heat map analysis in both modes showed that there were significant differences in metabolites between the two groups. Among 751 differential metabolites (DMs) detected in positive ion mode, 720 were up-regulated in the case group while 31 were down-regulated. In negative ion mode, 1891 DMs were detected, including 817 up-regulated metabolites and 1074 down-regulated metabolites in the case group. Analysis of differential metabolic pathways showed that the DMs of the two groups were mainly annotated and enriched in Biotin metabolism, N-Glycan biosynthesis, Taurine and hypotaurine metabolism, Pyrimidine metabolism, and Pentose and glucuronate interconversions. Conclusion: Significant differences exist in fecal metabolites between LBMJ infants and healthy controls. The study of differential metabolic pathways provides insights into the mechanism of LBMJ.

## Introduction

Jaundice, also known as hyperbilirubinemia, is a common clinical issue in newborns [1]. Neonatal hyperbilirubinemia occurs more frequently in breastfed infants compared to formula-fed infants [2]. Late-onset breast milk jaundice (LBMJ) is a type of jaundice that arises in neonates due to breastfeeding. It typically presents within the second week of life and is characterized by the abnormal accumulation of bilirubin, lasting 4–6 weeks or even 2–3 months. This prolonged elevation of bilirubin levels may lead to serious complications in newborn patients [3]. While LBMJ is generally considered to have a good prognosis, its impact on reduced breastfeeding rates and vaccine coverage has been widely observed in the community. This, in turn, may have adverse consequences for disease prevention and infant growth [1, 4]. The global prevalence of LBMJ has not been extensively documented, but research conducted in countries such as the United States Turkey and Taiwan has revealed that 20 to 30% of newborns exhibit symptoms of breast milk jaundice (BMJ) by the time they reach four weeks of age [5].

The etiology of LBMJ has been extensively researched, with a focus on the nutritional components and active factors present in breast milk. However, there is a lack of studies examining the intestinal flora and fecal metabolites in children with LBMJ.

In recent years, studies have demonstrated the involvement of intestinal microbiota in bilirubin metabolism, with the disruption of intestinal microbiota potentially leading to hyperbilirubinemia [6, 7]. Previous research conducted by our team identified a correlation between *Klebsiella* and LBMJ; however, the precise mechanism by which microbiota contributes to the development of LBMJ remains unclear [8]. The metabolism of both host and intestinal flora results in the production of numerous small molecules that may influence disease occurrence and progression by impacting metabolic pathways [9]. Metabolomics represents one of the most valuable methods for investigating the metabolism of small molecules, including short-chain fatty acids, vitamins, bile acids, hormones, and amino acids [10].

Recently, there has been a significant focus on the relationship between metabolism and jaundice in several studies. Alterations in metabolic profiles of jaundice-related diseases, particularly bile acid profiles, have been observed in both human and animal experiments [11–13]. As the field of metabolomics research in neonatology continues to advance, there is an increasing number of publications that concentrate on the correlation between metabolic changes associated with the extrauterine environment and metabolic diseases in early neonatal life [11].

So far, several metabolomic studies related to neonatal jaundice have identified various metabolites and metabolic pathways, but the conclusions have been inconsistent [14, 15]. In order to investigate the relationship between gut microbiota metabolites and LBMJ, metabolomics can be utilized to efficiently screen biomarkers and further analyze the molecular mechanism of LBMJ. Ultra-high performance liquid chromatography quadrupole time-of-flight tandem mass spectrometry (UHPLC-Q-TOF/MS) was employed for non-targeted determination of fecal metabolites in this study. A more comprehensive understanding of gut microbiome metabolomics may contribute to a better comprehension of the etiology of LBMJ, ultimately leading to improved prevention and treatment strategies.

## Methods

### Subjects

Patients with LBMJ and jaundice-free newborns from Peking University People’s Hospital were included in the study during October 1, 2020 to January 1, 2022 based on their participation in a follow-up mother-infant cohort.

Inclusion criteria were as follows:

- The mother is between 20 and 45 years old and has a medical record at Peking University People’s Hospital.
- Full-term infants born at Peking University People’s Hospital.
- Exclusive breastfeeding or predominantly breastfeeding (more than 80% breastfeeding).
- Presence of jaundice in the skin or sclera persisting for more than 3 weeks.
- Transdermal bilirubin value exceeding 7.87mg/dL on the 42nd day after birth.

Infants were excluded for the following reasons:

The infants were excluded if they had hemolytic disease, blood type incompatibilities, reticulocytosis, abnormal blood smear, polycythemia, a positive Coombs’ test, glucose-6-phosphate dehydrogenase deficiency, skull hematoma, hypothermia, intracranial hemorrhage, cholestasis, neonatal hypothyroidism, phenylketonuria screening that was positive and if they had used antibiotics or probiotics.

Finally, twelve infants with LBMJ and twelve healthy full-term newborns without exposure to antibiotics or probiotics after birth were included in the case group and the control group separately, based on the breast milk bank follow-up cohort established by our research group, which belongs to a prospective nested case-control study. The study received approval from the Ethics Committee of the Peking University People’s Hospital (Approval Number: 2020PHB113-01), and written informed consents were obtained from the guardians of all participants.

### Sample collection and storage

Newborn fecal samples are collected once at 42 days after birth without a specific time limit from October 1, 2020 to January 1, 2022. The specimens are obtained from diapers using sterile stool collection tubes, then promptly transferred to the laboratory and stored in a refrigerator at -80°C.

### Extraction and detection of fecal metabolites

Frozen fecal samples from all 24 neonates were utilized for metabolomics analysis. The extraction procedure was carried out according to Cheng K et al.21 and Ng JS et al. [16, 17], with some modifications. The fecal samples were ground into powder using liquid nitrogen, with a weight of 100 mg, and then mixed with 500 μL of pre-cooled ultra-pure water. The mixture was vortexed for 30 seconds, followed by centrifugation at 10,000 g for 15 minutes to obtain the supernatant, which was transferred to another centrifuge tube. Subsequently, 500 μL of pre-cooled methanol was added to the remaining sediment, swirled for 30 seconds, and centrifuged at 10,000 g for another 15 minutes. The two resulting supernatants were combined and allowed to stand at -20°C for a duration of 12 hours. Afterward, they were subjected to further centrifugation at 10,000 g for an additional period of time lasting up to15 minutes in order to obtain the final supernatant. This final supernatant was then passed through a filter membrane with a pore size of 0.22μm before being placed into sample bottles. The bottle caps were tightly sealed and wrapped with sealing film before storing them at -20°C until testing could be conducted. Ultra-high performance liquid chromatography quadrupole time-of-flight tandem mass spectrometry (UHPLC-Q-TOF/MS, Agilent Technologies, CA, USA) was utilized for the non-targeted determination of fecal metabolites. An Agilent Poroshell 120 SB-Aq column (100 mm × 2.1 mm i.d., 2.7 μm) was employed to separate the extracts. Quality control (QC) samples were prepared by mixing an equal volume of each sample (5 μL). The samples were used to evaluate the data reliability for metabolomics analysis. Both positive and negative ionization modes were utilized. The mobile phases A and B consisted of water and acetonitrile (0.1% formic acid), respectively. The gradient elution program was as follows: 0–2 min, 5% B; 2–20 min, linear increase from 5% to 100% B; 20–25 min, hold at 100% B; post-run time: 5min. The flow rate was set at a constant value of 300 μL/min, with an injection volume of 2 μL and a column temperature maintained at a steady level of 40°C.

For MS acquisition, an Agilent Q-TOF LC/MS system model number 6545 was employed with the following parameters: capillary voltage set at 4.0 kV for positive ionization mode and 3.5kV for negative ionization mode; gas temperature held constant at 300°C; drying gas flow rate maintained at 9 L/min; nebulizer pressure set to 35 psi; sheath gas temperature kept at 350°C; sheath gas flow rate adjusted to 11 L/min; mass range scanned from 50 to 1700 m/z.

### Statistical analysis

The Agilent Profinder software was utilized for retention time correction, peak identification, extraction, integration, alignment, and other data processing tasks on the original mass spectrum data, ultimately generating CEF files. Subsequently, the Agilent Mass Profiler Professional software was employed for statistical analysis, PLSDA and OPLSDA modeling under "supervised" conditions to evaluate the overall sample distribution. Material identification was conducted using the Metlin database, while the volcano plot was utilized to screen for differential compounds. Differential metabolite screening criteria were defined as metabolites with a Fold change > 2 and P value < 0.05 by t-test. MetaboAnalyst 6.0 and KEGG were applied for metabolite pathway analysis.

## Results

### Clinical characteristics of population

Based on previous research, 24 subjects were ultimately included in this study after excluding those with incomplete data. The study comprised of 12 LBMJ infants in the case group and 12 healthy individuals in the control group.

There were no significant differences observed between the two groups in terms of infants’ sex ratio, birth length, birth weight, 1 min Apgar score, 5 min Apgar score, as well as mothers’ BMI, maternal age, gestational week, delivery mode, parity and pregnancy complications (P>0.05) (Table 1).

**Table 1:** Clinical data of participants.

		LBMJ (n = 12)	Control (n = 12)	P value
Infants				
sex ratio (male/female)		1.00 (6/6)	0.50(4/8)	0.408
birth length (Mean±SD, cm)		49.83±1.70	49.67±1.67	0.811
birth weight (Mean±SD, g)		3256.67±391.62	3358.33±336.31	0.502
1 min *Apgar score (Mean±SD)		10±0	10±0	1.000
5 min *Apgar score (Mean±SD)		10±0	10±0	1.000
10 min *Apgar score (Mean±SD)		10±0	10±0	1.000
Mothers				
BMI (Mean±SD, kg/m2)		22.60±3.29	20.81±2.14	0.130
maternal age (Mean±SD, y)		33.33±2.64	31.58±3.29	0.165
gestational week (Mean±SD, w)		39.13±0.87	38.85±0.94	0.469
delivery mode (%)	Vaginal	10(83.30%)	9(75.00%)	0.615
	Cesarean	2(16.70%)	3(25.00%)	
**parity (%)	1	8(66.70%)	8(66.70%)	1.000
	2	4(33.30%)	4(33.30%)	
pregnancy complications (%)	Yes	2(16.70%)	5(41.70%)	0.178
	No	10(83.30%)	7(58.30%)	

*Apgar score represents birth health including suffocation.

**Data of parity represents times of delivery.

### Sample total ion flow chromatogram based on UHPLC-Q-TOF/MS

**Fig 1:**
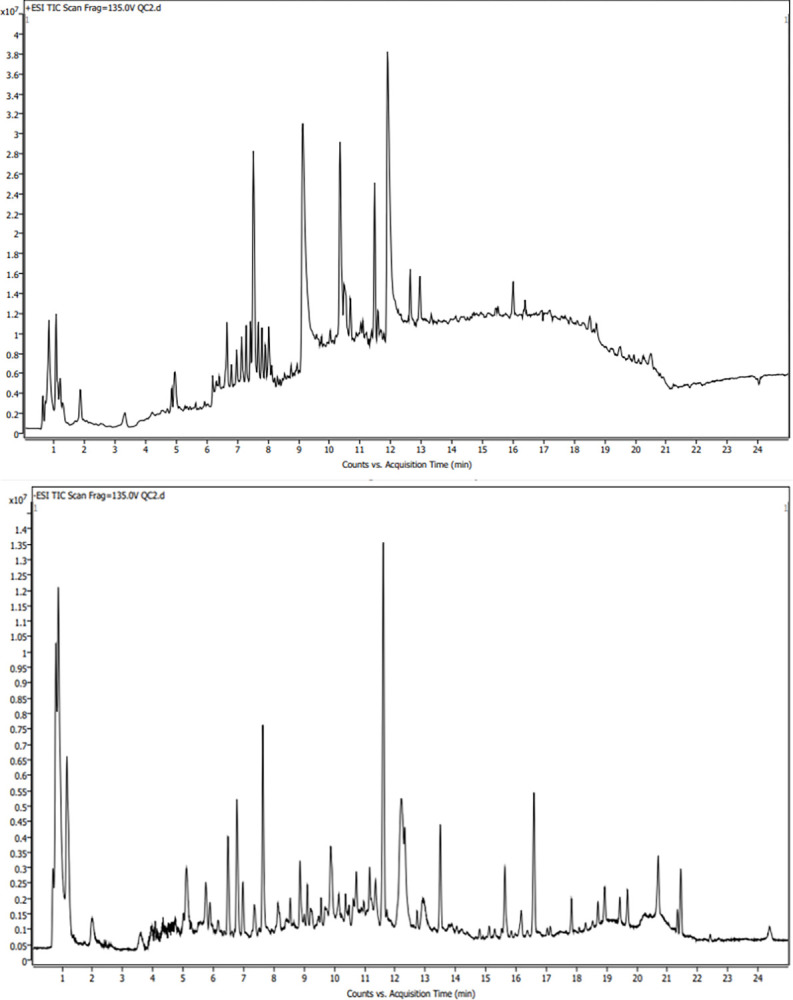
a. UHPLC-Q-TOF/MS TIC diagram of QC samples (positive ion mode). b. UHPLC-Q-TOF/MS TIC diagram of QC samples (negtive ion mode).

As depicted in Fig 1, metabolites in fecal samples were effectively separated under the positive and negative ion detection modes, with a retention time of less than 25 minutes.

### Principal components analysis (PCA) of case group and control group

**Fig 2:**
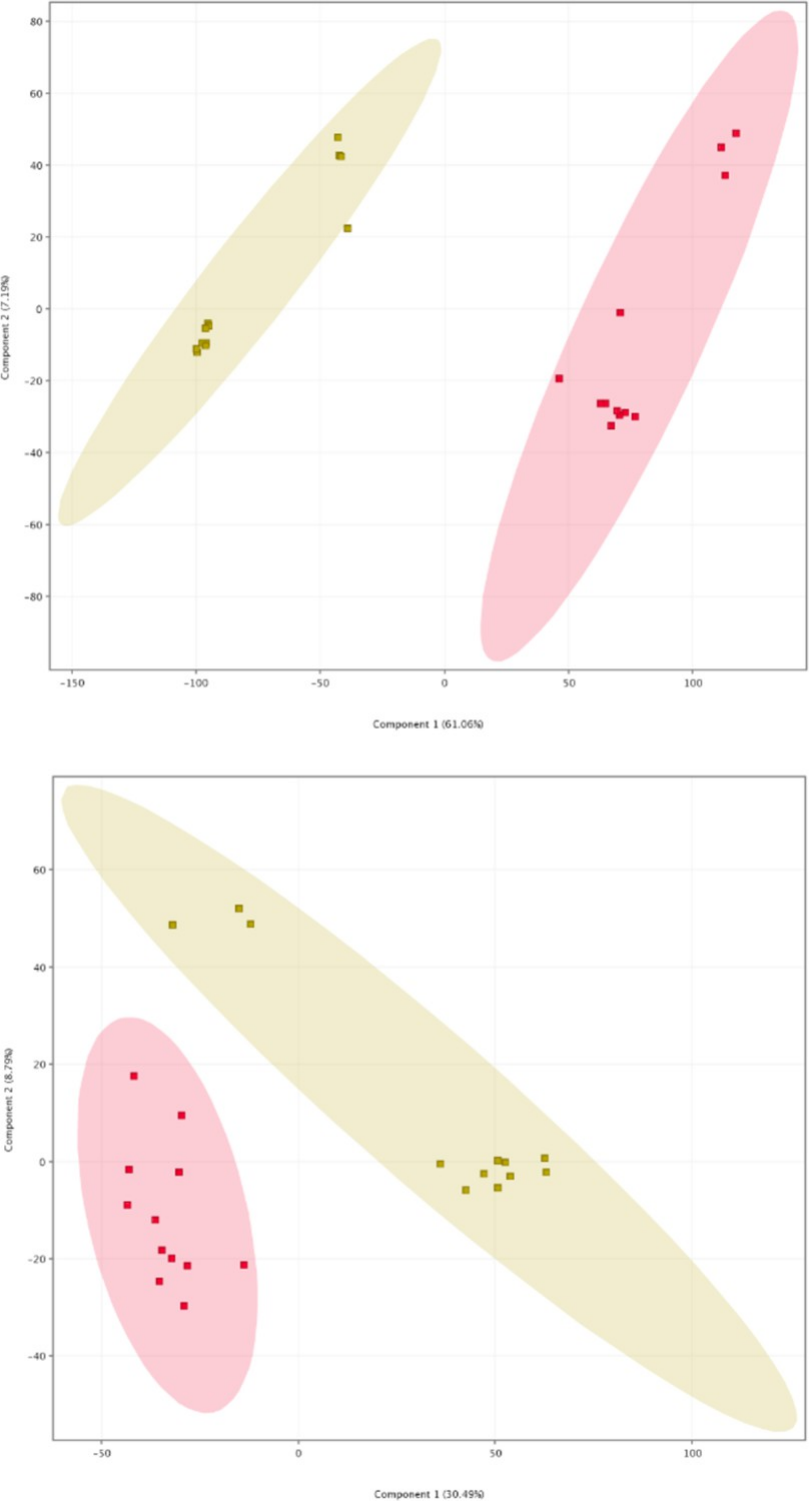
Two-dimensional PCA plot (a) positive ion mode; (b) negative ion mode. The yellow dots represent the case group and the red dots represent the control group.

PCA was conducted on mixed samples from the case and control groups in order to identify differences in metabolic profiles between them. The PCA plot of positive ion mode (Fig 2A) showed that samples from the case group were clustered together, positioned to the left of the control group samples. In the PCA plot of negative ion mode (Fig 2B), samples from the case group were grouped together, situated to the right of the control group samples. These findings suggest that our analysis was stable and reproducible.

### Cluster analysis of case group and control group

**Fig 3:**
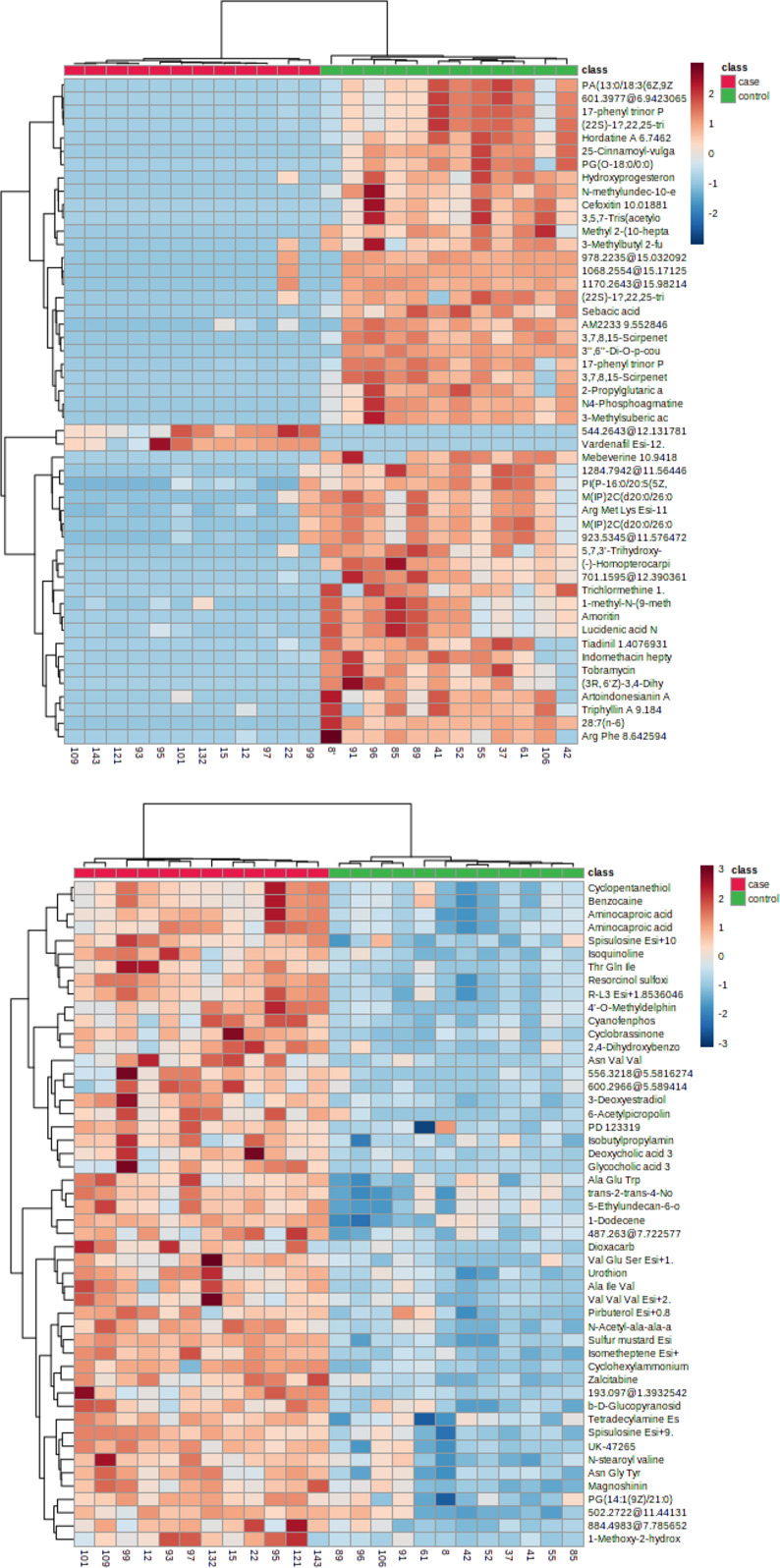
Heat maps of TOP 50 in (a) positive ion mode and (b) negative ion mode. The samples were grouped by horizontal coordinates, and the color scale from blue to orange indicated the content of differentially expressed metabolites from low to high.

Differences in the accumulation patterns of metabolites in the two groups of samples can be analyzed using heat maps (the top 50 from T-tests/ANOVA results are shown in Fig 3). The findings indicate significant differences in metabolite levels between the two groups. In the positive ion mode, the metabolites were categorized into two clusters. Metabolites in cluster 1 exhibited higher expression in the control group and lower expression in the case group. With only two exceptions in cluster 2, the majority of metabolites showed higher expression in the control group and lower expression in the case group. Conversely, in the negative ion mode, metabolites were divided into two clusters: both cluster 1 and cluster 2 displayed higher expression levels in the case group and lower expression levels in the control group.

### Differential metabolite analysis: Volcano maps

**Fig 4:**
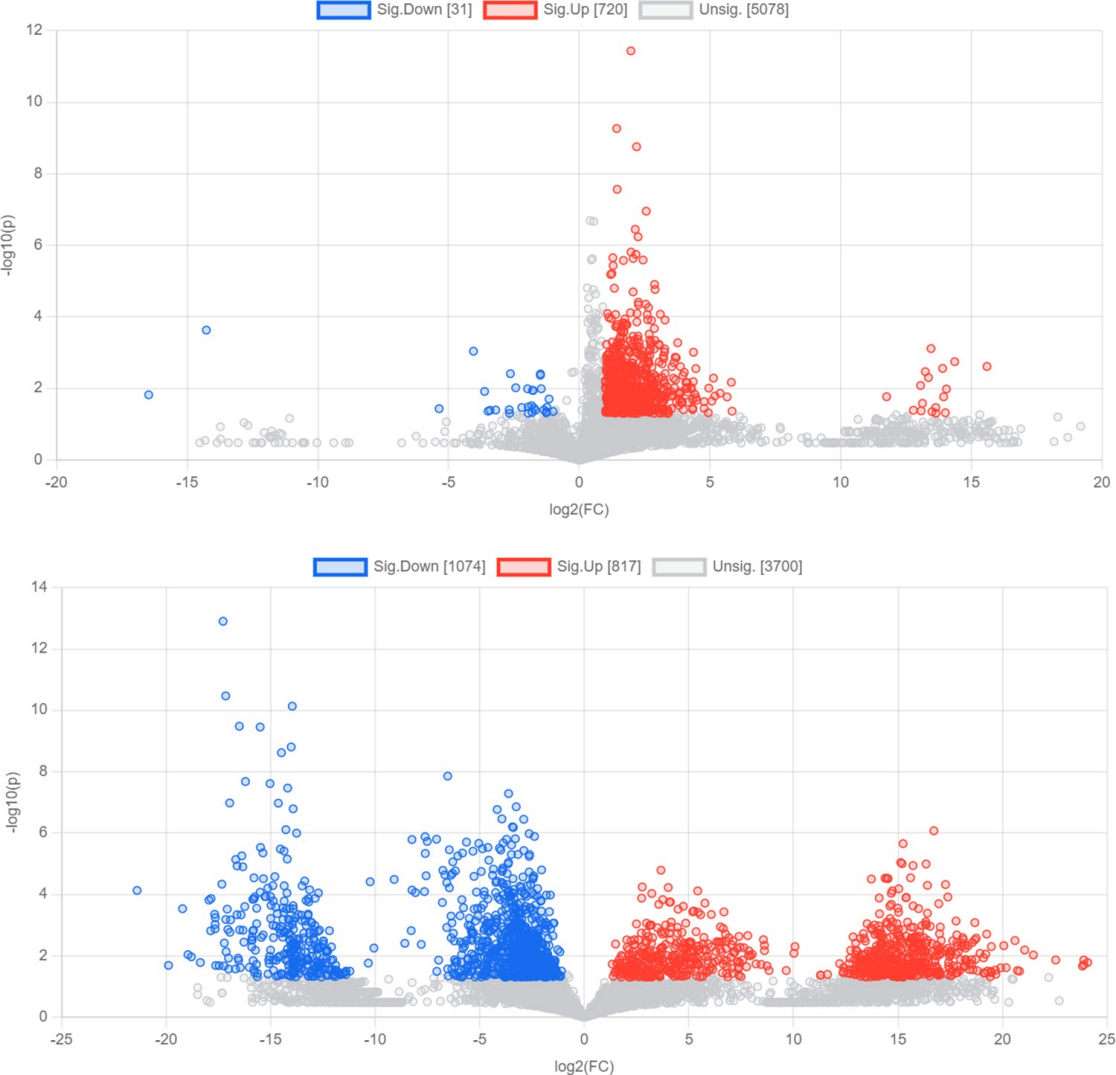
a. volcano plot in positive ion mode. b. volcano plot in negative ion mode.

Metabolites that satisfy both FC > 2 or < 0.05 and P < 0.05 simultaneously are considered as differential metabolites. In Fig 4A, there were a total of 720 up-regulated metabolites, indicating higher expression levels in the case group compared to the control group, and 31 down-regulated metabolites, indicating lower expression levels in the case group compared to the control group. In Fig 4B, there were a total of 817 up-regulated metabolites, indicating higher expression levels in the case group compared to the control group, and 1074 down-regulated metabolites, indicating lower expression levels in the case group compared to the control group.

### Analysis of differential metabolic pathways

The KEGG database was utilized for a comprehensive analysis of the pathways of differential metabolites. As indicated in Tables 2 and 3, 24 and 32 pathways were identified in the positive and negative ion modes, respectively. The total number of compounds in each pathway is presented in column 2; Hits represent the actual matched number from the data; Raw p denotes the original p-value calculated from the enrichment analysis; Impact refers to the pathway impact value calculated from pathway topology analysis.

**Table 2:** Differential metabolic pathways in positive ion mode.

KEGG pathway	Total number of compounds	Hits	Differential metabolites*	Raw p	-log10(p)	Impact
Nicotinate and nicotinamide metabolism	15	1	Nicotinate D-ribonucleoside	0.003813	2.4187	0
Caffeine metabolism	10	1	7-Methyluric acid	0.005382	2.2691	0
Purine metabolism	70	2	Inosine;8-Hydroxyadenine	0.006698	2.174	0.00955
Histidine metabolism	16	1	Imidazole-4-acetate	0.009499	2.0223	0
N-Glycan biosynthesis	41	2	Dolichyl phosphate; Dolichyl diphosphate;	0.014307	1.8445	0.10845
Arginine and proline metabolism	36	1	**Spermidine**	0.017808	1.7494	0.06628
Glutathione metabolism	28	1	**Spermidine**	0.017808	1.7494	0.00719
β-Alanine metabolism	21	1	**Spermidine**	0.017808	1.7494	0
Biotin metabolism	10	1	Biotinyl-5’-AMP	0.020436	1.6896	0.15
Arachidonic acid metabolism	44	1	11,12-EET	0.092701	1.0329	0.01474
Glycine, serine and threonine metabolism	33	1	Βine	0.26142	0.58266	0.05149
Tryptophan metabolism	41	1	Indole-3-acetaldehyde	0.28758	0.54124	0.0139
Pyrimidine metabolism	39	1	dTMP	0.29507	0.53007	0.08661
Linoleic acid metabolism	5	1	12(13)-EpOME	0.30567	0.51475	0
α-Linolenic acid metabolism	13	1	Stearidonic acid	0.30786	0.51165	0
Porphyrin metabolism	31	2	Bilirubin β-diglucuronide; Cob(II)yrinate a,c diamide	0.49906	0.30185	0.02795
Sphingolipid metabolism	32	4	Sphinganine; Globoside; GM3; Phytosphingosine	0.50413	0.29746	0.1159
One carbon pool by folate	9	1	**Dihydrofolate**	0.66725	0.17571	0.01587
Folate biosynthesis	27	2	THF-polyglutamate; **Dihydrofolate**	0.68781	0.16253	0.00434
Ubiquinone and other terpenoid-quinone biosynthesis	18	1	Ubiquinol	0.74079	0.13031	0
Pantothenate and CoA biosynthesis	20	1	Pantetheine 4’-phosphate	0.78493	0.10517	0.22449
Cysteine and methionine metabolism	33	1	4-Methylthio-2-oxobutanoic acid	0.81111	0.090922	0.06458
Drug metabolism—other enzymes	39	1	Irinotecan	0.8792	0.055913	0
Lysine degradation	30	1	Carnitine	0.92253	0.035018	0

* Recurring compounds are shown in bold.

**Table 3:** Differential metabolic pathways in negative ion mode.

KEGG pathway	Total number of compounds	Hits	Differential metabolites*	Raw p	-Log(p)	Impact
Lysine degradation	30	1	**L-Lysine**	5.93E-05	4.2272	0
Biotin metabolism	10	1	**L-Lysine**	5.93E-05	4.2272	0
Caffeine metabolism	10	2	1-Methyluric acid;5-Acetylamino-6-formylamino-3-methyluracil	0.001603	2.7952	0
Pyrimidine metabolism	39	4	Uridine;Deoxyuridine;Thymidine;Orotate	0.002098	2.6781	0.18316
Purine metabolism	70	2	Guanosine;Urate	0.003795	2.4207	0
Fatty acid degradation	39	1	**Octanoyl-CoA**	0.0058	2.2365	0.02355
Fatty acid elongation	39	1	**Octanoyl-CoA**	0.0058	2.2365	0.01148
Glycosaminoglycan biosynthesis—chondroitin sulfate / dermatan sulfate	10	1	Chondroitin	0.006707	2.1735	0
Pentose and glucuronate interconversions	19	2	**β-D-Glucuronoside**; L-Xylulose	0.007174	2.1442	0.10843
Metabolism of xenobiotics by cytochrome P450	68	2	7,12-Dimethylbenz[a]anthracene 5,6-oxide;1a,11b-Dihydro-4,9-dimethylbenz[a]anthra[3,4-b]oxirene	0.007319	2.1356	0.08163
Selenocompound metabolism	20	1	Dimethyl selenide	0.008723	2.0593	0
Ascorbate and aldarate metabolism	9	1	**β-D-Glucuronoside**	0.010438	1.9814	0
Primary bile acid biosynthesis	46	3	**Taurine**;3α,7α-Dihydroxy-5β-cholest-24-enoyl-CoA; 3α,7α,12α-Trihydroxy-5β-cholestanoate	0.012068	1.9184	0.07167
D-Amino acid metabolism	15	1	**D-Aspartate**	0.020155	1.6956	0
Taurine and hypotaurine metabolism	8	1	**Taurine**	0.022413	1.6495	0.42857
Alanine, aspartate and glutamate metabolism	28	2	**D-Aspartate; Citrate**	0.026877	1.5706	0
Citrate cycle (TCA cycle)	20	1	**Citrate**	0.026886	1.5705	0.09038
Glyoxylate and dicarboxylate metabolism	32	1	**Citrate;**	0.026886	1.5705	0.03175
Histidine metabolism	16	1	Imidazole-4-acetate	0.06201	1.2075	0
Starch and sucrose metabolism	18	1	**Sucrose**	0.073103	1.1361	0.05023
Galactose metabolism	27	1	**Sucrose**	0.073103	1.1361	0.03888
Biosynthesis of unsaturated fatty acids	36	1	Octadecanoic acid	0.10193	0.99169	0
Steroid hormone biosynthesis	87	2	Urocortisol; Tetrahydrocortisone	0.133	0.87615	0
Porphyrin metabolism	31	1	Bilirubin β-diglucuronide	0.25124	0.59991	0.02795
Linoleic acid metabolism	5	1	12(13)-EpOME	0.25994	0.58513	0
Pantothenate and CoA biosynthesis	20	1	N-((R)-Pantothenoyl)-L-cysteine	0.32818	0.48388	0
N-Glycan biosynthesis	41	1	Dolichyl phosphate D-mannose	0.32818	0.48388	0.00676
Folate biosynthesis	27	1	Dihydropteroate	0.35267	0.45264	0
Arginine biosynthesis	14	1	N-Acetyl-L-glutamate	0.55242	0.25773	0
Tryptophan metabolism	41	1	6-Hydroxymelatonin	0.6107	0.21417	0
Glycerophospholipid metabolism	36	1	Phosphatidylserin	0.61623	0.21025	0.04701
Terpenoid backbone biosynthesis	18	1	Farnesylcysteine	0.68944	0.16151	0

* Recurring compounds are shown in bold.

**Fig 5:**
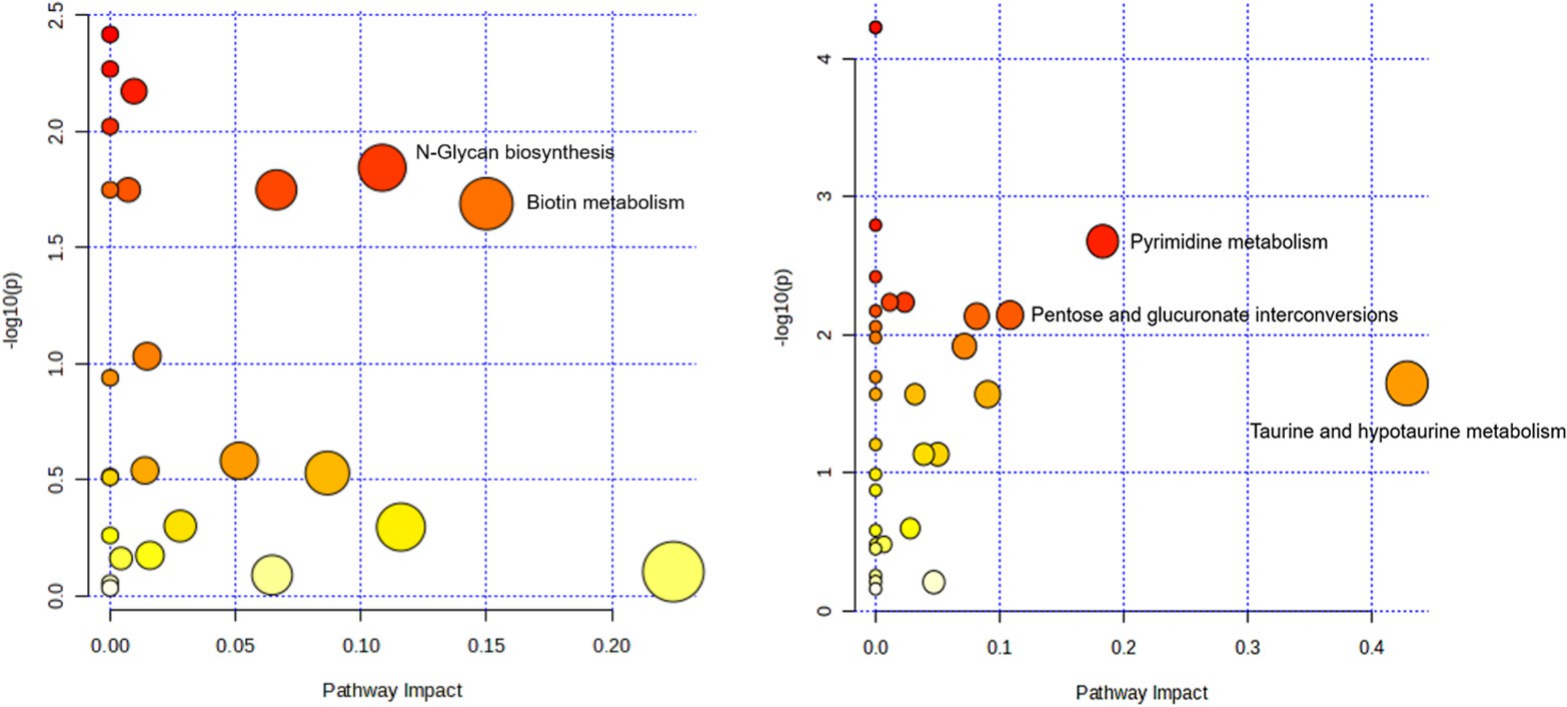
Summary of pathway analysis in (a) positive ion mode and (b) negative ion mode.

The pathway analysis in positive and negative ion mode was separately presented in Fig 5. The figure highlights important KEGG pathways, including Biotin metabolism, N-Glycan biosynthesis, Taurine and hypotaurine metabolism, Pyrimidine metabolism, and Pentose and glucuronate interconversions (Pathway Impact > 0.1, -log10(p) > 1.0).

## Discussion

Although an increasing number of studies have focused on LBMJ, the exact etiology of it has not been determined. Metabolomics can be utilized to detect and identify various metabolites in order to aid in the exploration of pathological mechanisms. In this study, we selected 24 LBMJ neonates based on strict metabolomic selection criteria to determine the changes in fecal metabolites and understand the pathogenesis of LBMJ.

The results of PCA and cluster analysis revealed that samples from the case group and the control group exhibited distinct separation into two different regions, indicating significant differences in the accumulation patterns of metabolites between the two groups. A recent study conducted by Yaxuan Li et al. utilized gas chromatography-mass spectrometry (GC/MS) to analyze fecal metabolites, and similarly observed a notable distinction in the metabolic profiles of infant fecal samples between the BMJ group and the healthy control group [18]. After comparing the differential metabolites, it was determined that in the positive ion mode, there were a total of 751 differential metabolites identified in both the case group and the control group. Among these, 720 metabolites were up-regulated and 31 metabolites were down-regulated. In the negative ion mode, a total of 1891 different metabolites were found in both groups, with 817 up-regulated metabolites and 1074 down-regulated metabolites. When compared to relevant studies utilizing targeted metabolomics, such as short-chain fatty acids and bile acids in infant feces, this study revealed a relatively large number of differential metabolites with more complex types [15, 18].

In addition, KEGG pathway analysis identified 56 differential metabolic pathways. The KEGG database is a powerful tool for metabolism analysis and metabolic network research, providing comprehensive information on metabolic pathways involving carbohydrates, nucleosides, amino acids, as well as genes, expression data, and metabolite content studied as a whole network [18, 19]. Based on the comprehensive analysis of P value and Pathway Impact, five main differential metabolic pathways were found between the case group and control group in this study (Fig 5). Representative differential metabolites include Biotinyl-5’-AMP, Dolichyl phosphate, Dolichyl diphosphate, Taurine, Uridine, Deoxyuridine, Thymidine,Orotate, β-D-Glucuronoside, and L-Xylulose. Furthermore, Spermidine, Dihydrofolate, L-Lysine, Octanoyl-CoA, β-D-Glucuronoside, Taurine, D-Aspartate, Citrate and Sucrose were identified multiple times in the pathways summary table (Table 2), indicating their potential significance in the metabolic pathways associated with LBMJ. These findings offer valuable insights for analyzing the metabolomics mechanism of LBMJ, and those differential metabolites mentioned above may be potential pharmacological targets and diagnostic analytes which may be clinical beneficial for LBMJ. Recent research on the etiology of BMJ has highlighted breast milk-related factors as predominant [20]. Breast milk is closely linked to the establishment of infant intestinal flora. The intestinal flora and its metabolites may impact bilirubin metabolism through participation in metabolic pathways, thereby influencing the onset and progression of jaundice. To date, limited studies have explored the fecal metabolomics mechanisms of BMJ.

The enhanced taurine and hypotaurine metabolism in the case group was predicted through untargeted metabolome analysis in our study. Taurine, identified as one of the most crucial differential metabolites in this study, suggests that it plays a vital role in bilirubin metabolism in infants with BMJ. The synthesis or metabolism of taurine is commonly observed in hepatocytes and is fundamental to the "enterohepatic circulation" of bile acids [21]. Despite its numerous diverse biological and important metabolic functions, its role in BMJ metabolomics mechanisms has yet to be fully elucidated. Previous studies have indicated that the concentration of taurine-conjugated bile acids in the stool of children with BMJ is significantly higher than that of healthy infants, and the proportion of serum taurine-conjugated bile acids increases proportionally to serum bilirubin levels [22, 23]. However, taurine has been documented to exhibit protective effects on neuronal cells against ischemia both in vivo and in vitro studies [24]. Recent research has also indicated that the enhancement of taurine and hypotaurine metabolism may have beneficial effects on the regulation of lipid metabolism and the restoration of liver function. This is achieved through its antioxidant and anti-inflammatory properties [25, 26]. These findings suggest the potential of taurine as an effective agent for preventing or treating neuronal damage in neonatal jaundice. Furthermore, they indicate that taurine and hypotaurine metabolism could be a targeted metabolic pathway for intervention therapy in bilirubin-induced neurological dysfunction.

Studies have indicated that taurine has the potential to regulate intestinal microecology by potentially inhibiting the growth of harmful bacteria, expediting the production of short-chain fatty acids, and reducing the concentration of LPS [27]. Additionally, research findings suggest that certain bacteria such as *Roseburia*, *Klebsiella*, *Clostridium IV*, and *Ruminococcaceae* may impact the development of metabolic diseases by influencing host metabolic pathways like taurine, sphingolipid, and ceramide [28]. Furthermore, a previous study revealed significant differences in the richness and diversity of intestinal microbiota between the LBMJ group and the control group. Notably, there was a marked increase in the dominant genus *Klebsiella* within the LBMJ group [8]. These findings suggest that *Klebsiella* may play a significant role in influencing intestinal homeostasis through certain metabolic pathways, ultimately leading to the development of hyperbilirubinemia. Recent research on bacterial sulfoacetaldehyde and taurine metabolism has revealed that IsfD, a reductase from *Klebsiella*, facilitates the reversible reduction of thioacetaldehyde to the corresponding isotaurine alcohol. This process is a crucial step in detoxifying carbonyl intermediates formed during bacterial nitrogen assimilation from the α-aminoalkanesulfonic acid taurine [29]. Therefore, it can be inferred that the pathogenesis of LBMJ may be linked to the interaction between *Klebsiella* and taurine metabolism.

Besides *Klebsiella*, which is one member of *Enterobacteriacea* in *phylum Proteobacteria*, *Firmicutes*, *Proteobacteria*, *Haemophilus*, *Escherichia*, *Morganella* and *Rothia* et.cl in infant intestine have also been found relevant with BMJ in several researches [30], and some specific gut microbiota, for example, *Lactobacillus* had been discovered that it had higher abundance of functional genes involved in taurine and hypotaurine metabolism in mice [31]. Even though the exactly relationship between metabolic pathways and intestinal flora of LBMJ infants is not clear, taurine and hypotaurine metabolism is probably closely related to fecal microbiota metabolism especially *Firmicutes* and *Proteobacteria* based on these recent studies [14, 32], thus it provides a future detective direction for us.

Another key differential metabolite identified in this study is β-D-glucuronoside, which plays a crucial role in the pentose and glucuronate interconversions pathway. A functional metabolomics study has shown that D-glucuronic acid is essential for binding unconjugated bilirubin via the pentose and glucuronate interconversions pathway [33]. The enzyme β-glucuronidase is pivotal in the "enterohepatic circulation", as it releases residual bilirubin in the intestine, allowing it to return to the liver through portal vein circulation for recombination [34]. Neonatal β-glucuronidase activity is significantly higher, and the intestinal wall is more permeable, resulting in higher overall unbound bilirubin concentrations and increased enterohepatic circulation. The expression of β-D-glucuronoside was significantly reduced in the case group of this study, suggesting that it may be decomposed under the action of more β-glucuronidase, possibly produced by an increase in *Klebsiella* [8]. Combining these results leads to a consistent conclusion: decreased intestinal β-D-glucuronoside levels in newborns may contribute to LBMJ occurrence by affecting the pentose and glucuronate interconversion pathway.

Other differentiated metabolites and metabolic pathways are primarily associated with amino acids, pyrimidines, vitamins, carbohydrates, and energy metabolism. Some of these have been confirmed to be linked to bilirubin metabolism in feces or serum or both. Previous studies have demonstrated that citric acid plays a role in the tricarboxylic acid cycle, which is beneficial for maintaining intestinal barrier function integrity and reducing the increase in enterohepatic circulation of bilirubin caused by damage [35, 36]. In this study, the concentration of citrate in the feces of the case group was lower than that of the control group, potentially leading to slower intestinal development and energy synthesis in infants, resulting in bilirubin accumulation and BMJ occurrence. Aspartic acid is involved in uridine diphospho-glucuronosyltransferase (UDPGT) synthesis, a rate-limiting enzyme during bilirubin metabolism. Gourley et al. [37] found that supplementation with aspartic acid could reduce transcutaneous bilirubin levels, promote excretion of fecal bilirubin, and alleviate jaundice. In this study, the fecal metabolite D-aspartate content was lower in infants from the case group compared to those from the control group; this may lead to a deficiency in UDPGT synthesis due to aspartic acid shortage and slow down bilirubin metabolism resulting in its accumulation. Spermidine is crucial for regulating cell growth and differentiation while preventing liver damage and promoting liver tissue regeneration [38]. The down-regulated fecal spermidine content observed in the case group may lead to jaundice due to insufficient metabolic capacity for bilirubin caused by immature liver function. In addition, strong relationship between bilirubin metabolism and some metabolites in blood samples, including Taurine, Citrate and Sucrose, which were found to be significant in this study, has been discovered in several studies [39–41], indicating that there may be some correlations of these key metabolites from different sources of specimens.

It is well-known that the gut microbiome plays a central role in the homeostasis of bile acid metabolism [42], and the present study has shown that there are several differential metabolites related to specific pathways in which some type of intestinal microbiota may play essential roles. The composition and activity of the gut microbiota codevelop with the host and is subject to a complex interplay that depends on the host genome, nutrition, and life-style [43]. While conjugated bile acids are deconjugated by bile salt hydrolases produced by gut microbiota, bile acids in turn have direct antimicrobial effects on gut microbiota and indirect effects through FXR-induced antimicrobial peptides, and decrease of bile acid levels in the gut favors the growth of Gram-negative bacteria [44]. Therefore, variations in microflora and fauna may affect the disorder and vice-versa, and deeper exploration about the causal relationship between them should be performed in the near future.

There are two strengths in this study, namely the unique scheme about fecal metabolism of LBMJ utilizing untargeted metabolomics approach while few studies had focused on it, and the novel differential metabolites discovered which may provide new possible molecular mechanism and clinical strategy of LBMJ. However, the study has two drawbacks. First, in this study, there was a lack of exploration of the influence of maternal factors, especially breast milk composition, maternal diet and other lifestyle factors, on infant intestinal metabolites, because LBMJ is inevitably associated with breast milk which is related with various maternal factors. Second, this is a cross-section study with a small number of subjects. Though subjects had been selected through rigorous criteria and significant differences were observed in this study after metabolomics statistical analyses, the data might not accurately reflect the characteristics of a broader, more diverse population. Thus deeper exploration will take place as the size of mother-infant cohort extends and more maternal factors are taken into account.

## Conclusion

Given the high prevalence of LBMJ and its potential impact on infant health, this study is dedicated to exploring the molecular mechanisms underlying the metabolomics aspects of the disease and discovered that there are significant differences in fecal metabolites between infants with LBMJ and healthy controls. The discovery of differential metabolic pathways provides novel insights into the mechanism of LBMJ which may be related to the regulation of infant metabolism especially bilirubin metabolism by influencing the levels of taurine, β-D-glucuronoside, citrate, D-aspartate, thymidine, as well as other metabolites mentioned above in infant intestine, although they were first discovered in this study. This study provides theoretical support for the intestinal metabolomics mechanism of LBMJ, and suggests that intestinal flora and its metabolites may be used as biomarkers for diagnosing and monitoring the severity of LBMJ in the future. Moreover, this study also provides theoretical and experimental basis for the regulation of intestinal flora and its metabolites, which is also a new direction for the treatment strategy of LBMJ.

## Supporting information

S1 Data Detail data of DMs with FC and P-value in positive ion mode and negative ion mode. (XLSX)
